# Maximal exercise and erythrocyte fatty‐acid status: a *lipidomics* study

**DOI:** 10.14814/phy2.14040

**Published:** 2019-04-24

**Authors:** Benjamin Gollasch, Inci Dogan, Michael Rothe, Maik Gollasch, Friedrich C. Luft

**Affiliations:** ^1^ Experimental and Clinical Research Center (ECRC) A Joint Institution of the Charité Medical Faculty and Max Delbrück Center (MDC) for Molecular Medicine Berlin Germany; ^2^ HELIOS Klinikum Berlin‐Buch Berlin Germany; ^3^ LIPIDOMIX GmbH Berlin Germany

**Keywords:** Erythrocytes, exercise, fatty acids, lipidomics

## Abstract

Omega‐3 fatty acids have long been ascribed a positive cardiovascular function. However, the plasma measurements invariably ignore 40% of the blood specimen, cells that engage in continuous exchange with their environment. In our study, we included all components of the circulating blood. Erythrocyte or red‐blood‐cell (RBC) *n*−3 fatty acid status has been linked to cardiovascular disease and death. A low omega‐3 index is an independent risk factor for cardiovascular disease and mortality. We tested the hypothesis that acute, maximal exercise would influence the relationship between RBC and serum fatty acids. RBC fatty acids profiling was achieved using targeted HPLC‐MS mass spectrometry. Healthy volunteers performed maximal treadmill exercise testing using the modified Bruce protocol. Central hemodynamics were monitored and maximal workload was assessed in metabolic equivalents (METs). Venous blood was obtained for RBC lipidomics. With the incremental exercise test, no fatty acid‐level variations were found in RBCs, while heart rate and arterial blood pressure increased significantly. No changes occurred in the omega‐3 quotient, namely the percentage of eicosapentaenoic acid and docosahexaenoic acid in RBC fatty acids in the RBC membrane. Nonetheless, maximal (13.50 ± 1.97 METs) exercise intensity led to a decrease of RBC lauric acid (C12:0) in the recovery period. These data suggest that despite significant hemodynamic effects, short‐term maximal exercise is insufficient to alter RBC *n*−3 and other fatty‐acid status, including the omega‐3 quotient, in healthy individuals. RBC lauric acid deserves further scrutiny as a potential regulator of cardiovascular and metabolic functions.

## Introduction

**Figure 1:**
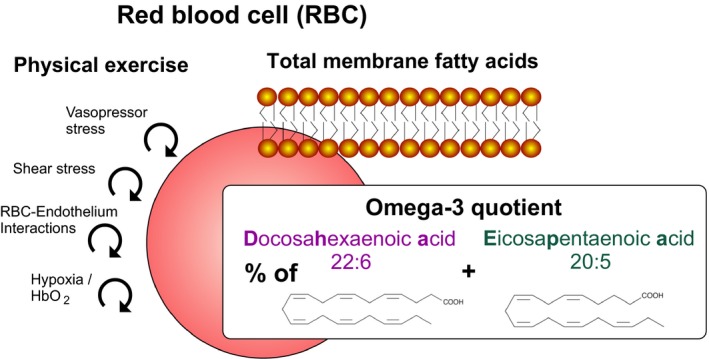
Schematic illustration of the hypothetic influence of short‐term maximal exercise associated with increased vasopressor and shear stress, red blood cell (RBC)‐endothelial interactions, and tissue hypoxia facilitating release of oxygen from RBCs on relative EPA+DHA content in RBC, i.e. omega‐3‐ quotient, which is the percentage of EPA+DHA in red cell fatty acid lipids.

Erythrocyte or red‐blood‐cell (RBC) *n*−3 fatty‐acid status is related to health outcomes, including cardiovascular disease, myocardial infarction, arrhythmias, and sudden death (Bucher et al. 2002). Fish consumers have less cardiovascular disease (Huang et al. 2011; InterAct Consortium, 2011). The two most important omega‐3 (*n*−3) polyunsaturated fatty acids are eicosapentaenoic acid (EPA, C20:5 *n*−3) and docosahexanoic acid (DHA, C22:6 *n*−3). DHA‐rich fish oil supplementation favorably modulates body composition in type‐2 diabetic and obese patients (Mansoori et al. 2015). *n*−3 fatty‐acid affect diets RBC membrane fatty acid composition (Popp‐Snijders et al. 1986). For example, a daily fish‐oil concentrate supplement, providing 3 g of omega‐3 fatty acids, increases incorporation of C20:5 omega 3 into RBCs, at the expense of C18:2 omega 6 fatty acids, but total unsaturation of phospholipids is increased (Cartwright et al. 1985). The *n*−6 fatty acid, linoleic acid (LA), and the *n*−3 fatty acids, linolenic acid (ALA), eicosapentaenoic acid (EPA), and docosahexaenoic acid (DHA) collectively protect against coronary heart disease (Wijendran and Hayes 2004). The *n*−3 fatty acids, especially EPA and DHA, are potent anti‐arrhythmic agents (von Schacky 2004; Wijendran and Hayes 2004). Patients with low RBC *n*−3 and *n*−6 fatty acids, palmitoleic acid, and stearic acid status have an increased risk of acute coronary syndromes (Shearer et al. 2009). EPA and DHA improve endothelial function, lower blood pressure, and favorably act on platelets (Wijendran and Hayes 2004). A low omega‐3 index (the percentage of EPA+DHA in red‐cell lipids) independently increases cardiovascular‐disease risk and mortality (von Schacky 2015; Kleber et al. 2016a,2016b; Thuppal et al. 2017). RBC fatty‐acid status is particularly important for assessing disease risk prediction (Kim et al. 2018). During exercise, triacylglycerides, an energy reservoir in adipose tissue, are hydrolyzed to free fatty acids, which are then released to the circulation, providing a fuel for working muscles (Mika et al. 2019). The most consistently observed effect has been an increase in the relative amount of unsaturated, especially monounsaturated, non‐esterified fatty acids in plasma after acute exercise, such as running, cycling, or swimming of moderate intensity for 0.5–2 h per bout (Nikolaidis and Mougios 2004). These exercise‐related changes in fatty acid metabolism in adipose tissue and circulating blood could contribute to beneficial cardiovascular and metabolic effects of physical activity. We tested the hypothesis that acute, maximal exercise would influence the relationship between RBC and serum fatty acids (Fig. 1).

## Methods

The Charité University Medicine institutional review board on the use of humans in research approved the study and written informed consent was obtained. The study was duly registered: ClinicalTrials.gov Identifier: NCT03121885, https://clinicaltrials.gov/ct2/show/NCT03121885. Recruitment was primarily *via* person‐to‐person interview. Prior to participation in the study, six healthy volunteers (five male and one female) signed informed consent forms which outlined the procedures to be taken and the possible risks involved. The age of the subjects was 38 ± 15 years. The body mass index of the individuals was 27.9 ± 6.6 kg/m^2^. All subjects were non‐trained. They were not taking medications. Following a routine physical examination at baseline levels, each subject underwent a maximal treadmill Bruce test, which is recommended by guidelines for ergometry of the German Society of Cardiology (Bruce et al. 1973; Trappe and Lollgen 2000). The test was preceded by 2 × 3 min warm up periods (stages 1 and 2 of the Bruce protocol) during which treadmill speed was maintained at a constant speed of 2.7 km/h and at zero or 5% grade. Treadmill speed and grade were then increased at three min intervals to a maximum of 20% grade at 8.8 km/h. Workload was assessed in metabolic equivalents (METs) from 5–18 METS. The test was terminated when the subjects informed the investigator that they could no longer proceed.

**Figure 2:**
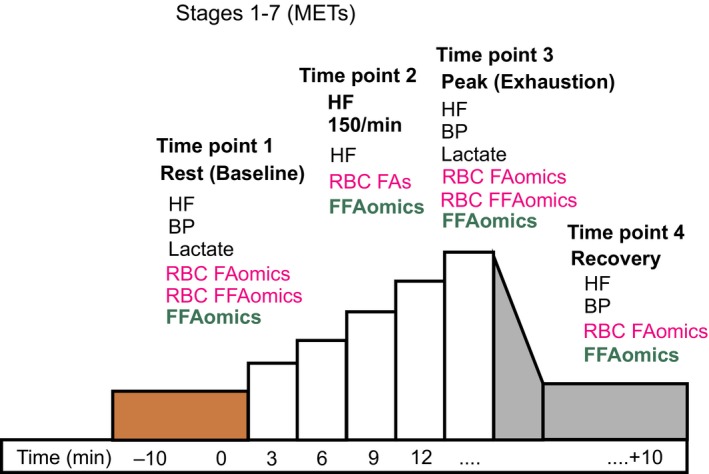
Schematic illustration of the experimental protocol used. HF, heart rate; BP, blood pressure; RBC, red blood cell; FFA, free fatty acid; FA, fatty acid; FAomics; plasma fatty acid lipidomics. METs, metabolic equivalents of task.

Heart rates were monitored continuously by heart‐rate monitor worn around the subject's torso (Polar T31, Polar Electro, Kempele, Finland) throughout the tests. Arterial pressure was measured in each subject while sitting prior to the exercise test (−10 min), after termination of the test (exhaustion), and 10 min recovery after the end of the running test *via* a sphygmomanometer (Critikon, Inc., Johnson & Johnson, New Jersey), which comprised an inflatable (Riva‐Rocci) cuff placed around the upper arm. Venous blood was collected from a catheter placed in a contralateral forearm vein (i.e. the antecubital vein) of each subject in the sitting position prior to the exercise test (−10 min), after termination of the test (exhaustion), and 10 min recovery after the end of the running test (Fig. 2). An additional blood sample was collected in each subject during running when the heart rate reached 150 beats per minute. We did not measure blood pressure at this time point (HF 150) because valid blood pressure measurements could not be obtained during running using the above sphygmomanometer. All samples were analyzed for RBC total fatty acids status and plasma free fatty acids. RBC free fatty acid status was determined at rest and at maximal workload. RBCs were separated from EDTA blood by centrifugation and total fatty acids in RBCs or plasma were determined by liquid chromatography mass (HPLC‐MS) spectrometry described in (Fischer et al. 2014). Serum lactate was determined in blood samples obtained from ear lobe at rest and at maximal workload.

We performed sample size calculation for a difference in means in omega‐3 quotients. We found that our study would require a sample size of 5 (number of pairs) to achieve a power of 80% and a level of significance of 5% (two sided), for detecting a mean of the differences of 0.021 (Harris et al. 2017) between pairs, assuming the standard deviation of the differences to be 0.88 (Fischer et al. 2014). Thus, in our sample size calculation, statistical significance and clinical significance both were taken into account.

Descriptive statistics were calculated and variables were examined for meeting assumptions of normal distribution without skewness and kurtosis. In order to determine statistical significance between the trials at the various time intervals, one‐way repeated measures analysis of variance (ANOVA) was conducted and the 0.05 level of significance (*P*) was chosen. The analysis included Mauchly's test of sphericity followed by applying the test of within subjects’ effects with Greenhouse–Geisser correction to ensure sphericity assumption. When significant differences were found, Tukey's honestly‐significant‐difference post‐hoc test was used for pairwise comparisons. Planned hypotheses (one‐tailed or two‐tailed paired *t*‐tests as appropriate) were tested to follow up the initial ANOVA findings. All data are presented as mean ± SD. All statistical analyses were performed using SPSS Statistics software (IBM Corporation, Armonk, NY) or All‐Therapy statistics beta (AICBT Ltd, Vancouver, Canada).

## Results

The hemodynamic and lactate values (Table 1) confirmed maximal exertion. The values include measurements prior to the exercise test (−10 min, rest), after termination of the test (exhaustion), and 10 min after the end of the running test (recovery period). An additional blood sample was collected in each subject during running when the heart rate reached 150 beats per minute. Exercise induced an increase in both heart rate and systolic blood pressure, which normalized to baseline (resting) levels in the recovery period. All individuals terminated exercise at a maximal workload of 13.50 ± 1.97 METs (Bruce stages 6.33 ± 0.82). Our ANOVA analysis showed that there were differences between heart rate and systolic blood pressure at the different time points: rest (baseline), exhaustion and recovery (Table 1). At this maximal workload, heart rate, systolic and diastolic blood pressure increased from 71 (baseline) to 185 beats per min from 135 to 190 mmHg and from 81 to 91 mmHg respectively (*P *<* *0.0001 each, Tukey's honestly‐significant‐difference post‐hoc tests, Table 1). Lactate levels were determined at rest and exhaustion. Lactate levels increased from 1.38 ± 0.30 mmol/L to 9.49 ± 2.10 mmol/L (*P *<* *0.0001, *t*‐test, Table 1).

**Table 1:** Effects of exercise on hemodynamics and lactate (means ± SD, *n* = 6)

Parameter	Time point 1 (rest)	Time point 2 (HF 150)	Time point 3 (exhaustion)	Time point 4 (recovery)	Mauchly's test, *P* value	Greenhouse–Geisser, *P* value	*t*‐Test, *P* value
Heart rate (beats per min)	71 ± 10	150	185 ± 6	94 ± 11	<0.001	<0.001	
Systolic arterial blood pressure (mm Hg)	135.3 ± 9.1	n.d.	190.3 ± 16.6	127.5 ± 13.1	<0.001	<0.001	
Diastolic arterial blood pressure	81.2 ± 14.4	n.d.	90.7 ± 16.4	76.3 ± 10.2	0.065	0.097	
Lactate (mmol/L)	1.38 ± 0.30	n.d.	9.49 ± 2.10	n.d.			<0.001

Since the impact of acute exercise on individual RBC fatty acids is unknown, we used an explorative statistical approach on measured fatty acid profiles, ranging from C12:0 to C22:6 *n*−3 fatty acids (Table 2), at the different time points: rest (baseline), the time when heart rate reached 150 beats per min, exhaustion and recovery. Our ANOVA analysis showed that there were no differences between the individual RBC fatty acids of all four groups. Our ANOVA analysis also showed that there were also no differences between omega‐3 quotients of all four groups (Table 3). The RBC omega‐3 quotients at rest and exhaustion were 0.067and 0.063 respectively (*n* = 6, *P *=* *0.148, *t*‐test). The data indicate that the short‐term maximal exercise is insufficient to alter RBC fatty acid status repeatedly measured during maximal exercise and post exercise periods in healthy individuals, including omega‐3 quotients.

**Table 2:** Lipidomics of RBCs in response to exercise (*n* = 6)

Fatty acid (*μ*g/g)	Time point 1 (rest)	Time point 2 (HF 150)	Time point 3 (exhaustion)	Time point 4 (recovery)	Mauchly's test, *P* value	Greenhouse–Geisser, *P* value
C12:0	1.033 ± 1.677	2.060 ± 2.505	1.638 ± 1.837	0.102 ± 0.250	0.411	0.296
C14:0	16.296 ± 12.774	16.147 ± 2.943	23.435 ± 10.156	14.890 ± 8.277	0.234	0.271
C14:1	0.965 ± 0.230	1.048 ± 0.122	1.278 ± 0.448	1.176 ± 0.413	0.079	0.201
C16:0	551.129 ± 158.224	630.805 ± 160.504	601.531 ± 117.900	526.699 ± 101.248	0.512	0.355
C16:1	16.427 ± 6.420	17.171 ± 2.651	21.676 ± 5.304	17.686 ± 5.453	0.622	0.248
C18:0	349.925 ± 102.151	441.744 ± 156.675	379.324 ± 108.094	326.366 ± 79,891	0.258	0.202
C18:1 cis	369.359 ± 109.595	372.508 ± 83.028	386.719 ± 49.709	352.496 ± 70.850	0.784	0.660
C18:1 trans	11.990 ± 3.208	13.388 ± 3.860	14,371 ± 38042	12,703 ± 28,339	0.110	0.444
C18:2	378,376 ± 1172,868	382.639 ± 72.9316	420.356 ± 52.0496	371.114 ± 82.9781	0.940	0.462
C18:3 *n*−3	8.298 ± 2.8805	8.663 ± 2.2216	10.110 ± 2.5471	8.355 ± 1.9315	0.541	0.138
C18:3 *n*−6	3.436 ± 1.1632	3.693 ± 1.009	4.287 ± 1.090	3.521 ± 0.630	0.981	0.145
C20:1	12.580 ± 4.273	11.799 ± 2.585	12.157 ± 1.821	10.424 ± 2.448	0.620	0.295
C20:3	73.136 ± 32.1532	65.684 ± 23.583	68.295 ± 21.397	61.948 ± 24.819	0.193	0.264
C20:4 *n*−3	2.538 ± 1.449	2.355 ± 1.189	2.561 ± 1.185	2.213 ± 1.160	0.442	0.262
C20:4 *n*−6	442.260 ± 116.511	465.333 ± 85.263	459.271 ± 56.357	431.627 ± 87.459	0.149	0.629
C20:5 *n*−3 (EPA)	21.916 ± 5.707	23.876 ± 5.821	24.717 ± 3.954	21.794 ± 3.076	0.432	0.391
C22:1	0.810 ± 0.329	0.873 ± 0.282	0.776 ± 0.178	1.307 ± 1.033	0.001	0.345
C22:5 *n*−3	29.426 ± 5.518	29.755 ± 3.613	30.117 ± 4.364	27.642 ± 3.240	0.288	0.597
C22:5 *n*−6	7.454 ± 2.019	7.277 ± 1.107	7.362 ± 1.287	6.858 ± 1.664	0.398	0.566
C22:6 *n*−3 (DHA)	145.677 ± 57.864	137.331 ± 42.755	138.461 ± 34.927	129.536 ± 48.290	0.631	0.329

**Table 3:** Omega‐3 quotient of RBCs in response to exercise (means ± SD, *n* = 6)

Fatty acid (*μ*g/g)	Time point 1 (rest)	Time point 2 (HF 150)	Time point 3 (exhaustion)	Time point 4 (recovery)	Mauchly's test, *P* value	Greenhouse–Geisser, *P* value
C20:5 *n*−3 (EPA) + C22:6 *n*−3 (DHA)	167.593 ± 63.295	161.207 ± 46.482	163.179 ± 36.480	151.330 ± 50.757	0.65	0.42
Total fatty acids	2443.031 ± 693.691	2634.149 ± 535.512	2608.440 ± 343.774	2328.457 ± 445.098	0.65	0.42
[C20:5 *n*−3 (EPA) + C22:6 *n*−3 (DHA)] / Total fatty acids	0.0670 ± 0.0087	0.0610 ± 0.0119	0.0631 ± 0.0129	0.0641 ± 0.0135	0.77	0.09

High levels of oleic acid (OA, C18:1 cis), palmitic acid (C16:0), palmitoleic acid (C16:1), and alpha‐linolenic acid (C18:3 *n*−3) in RBCs have been found to associate with all‐cause and cardiovascular mortality (Delgado et al. 2017) or sudden cardiac death (Lemaitre et al. 2009, 2010). Our study revealed no change in levels of fatty acids C16.0, C16:1, C18:1, and C18:3 *n*−3 in RBCs at maximal exercise (for all *P *>* *0.05, *t*‐tests).

We next focused on lauric acid (C12:0), which accumulates in blood under exhaustive exercise or in ischemia (Di Paola and Lorusso 2006) and is an “exchangeable” or “reversibly‐bound” free fatty acid in RBCs (Spector et al. 1972) belonging to medium‐chain fatty acids preferentially oxidized in mitochondria in comparison with long‐chain fatty acids (Di Paola and Lorusso 2006). We therefore hypothesized that RBC lauric acid levels at maximal exercise will decrease in the recovery period. Our results show that RBC lauric acids at exhaustion decreased from 1.638 ± 1.837 to 0.102 ± 0.250 *μ*g/g in the recovery period (*n* = 6, *P *=* *0.035, *t*‐test). This effect was associated with a reduction of heart rate from 185 ± 6 to 94 ± 11 beats per minute (*P *<* *0.001, Tukey's honestly‐significant‐difference post‐hoc test). It was also associated with a decrease of systolic and diastolic blood pressures from 190.3 ± 16.6 to 127.5 ± 13.1 mmHg and from 90.7 ± 16.4 to 76.3 ± 10.2 mmHg, respectively (*P *<* *0.001 each, Tukey's honestly‐significant‐difference post‐hoc tests), which indicates possible involvement of this fatty acid in hemodynamic and metabolic recovery after short‐term maximal exercise.

To provide insights into possible mechanisms underlying effects of exhaustive exercise on individual RBC fatty acids status, we measured circulating plasma free fatty acids (Table 4). The results show that plasma levels of the free fatty acids C16:0, C16:1, C18:0, C18:1 cis, and C18:2 are relatively high, whereas levels of C12:0, C14:1, C18:3 *n*−6, C20:4 *n*−3, and C22:1 are relatively low. Besides lauric acid (C12:0), none of the circulating free fatty acids did change during exercise and post exercise. We also measured free fatty acids in RBCs (Table 5). The results show that free levels of fatty acids in RBCs are very low, i.e. ~1–2% of the respective individual total RBC fatty acids. None of the free fatty acids in RBCs did change during exercise (Table 6). Post exercise circulating plasma level of lauric acid (10.623 ± 11.010 *μ*g/mL) did not differ from circulating levels measured at exhaustion (9.542 ± 10.393 *μ*g/mL, *n* = 6, *P *=* *0.081, *t*‐test).

**Table 4:** Circulating plasma free fatty acids in response to exhaustive exercise (*n* = 6).

Free fatty acid (*μ*g/mL)	Time point 1 (rest)	Time point 2 (HF 150)	Time point 3 (exhaustion)	Time point 4 (recovery)	Mauchly's test, *P* value	Greenhouse–Geisser, *P* value
C12:0	8.823 ± 9.831	9.168 ± 8.978	9.542 ± 10.393	10.623 ± 11.010	0.316	0.413
C14:0	44.312 ± 10.303	47.071 ± 14.788	49.479 ± 8.381	51.962 ± 13.056	0.154	0.397
C14:1	2.621 ± 0.964	2.702 ± 1.171	2.515 ± 0.575	3.006 ± 1.039	0.270	0.463
C16:0	331.130 ± 41.900	341.226 ± 25.159	347.013 ± 47.183	373.464 ± 42.320	0.687	0.182
C16:1	40.836 ± 10.314	41.121 ± 6.758	39.598 ± 10.293	42.831 ± 10.876	0.623	0.676
C18:0	150.108 ± 23.790	156.811 ± 11.718	157.947 ± 25.934	171.468 ± 22.982	0.818	0.160
C18:1 cis	283.376 ± 44.731	284.895 ± 25.185	282.537 ± 50.712	306.409 ± 39.356	0.634	0.360
C18:1 trans	21.296 ± 9.985	21.123 ± 6.187	22.490 ± 9.685	24.338 ± 8.694	0.177	0.447
C18:2	328.375 ± 59.582	343.606 ± 22.969	333.085 ± 68.081	366.570 ± 46.221	0.588	0.415
C18:3 *n*−3	10.732 ± 2.374	11.089 ± 3.406	10.636 ± 3.306	11.823 ± 2.722	0.440	0.522
C18:3 *n*−6	5.223 ± 2.052	5.201 ± 1.484	4.922 ± 1.967	5.263 ± 1.451	0.242	0.767
C20:1	10.330 ± 2.172	10.007 ± 2.427	8.119 ± 4.589	10.717 ± 2.580	0.229	0.227
C20:3	50.696 ± 25.737	52.777 ± 28.062	51.191 ± 32.891	55.044 ± 33.320	0.004	0.617
C20:4 *n*−3	2.370 ± 1.219	2.571 ± 1.242	2.583 ± 1.561	2.674 ± 1.441	0.158	0.338
C20:4 *n*−6	139.153 ± 28.757	147.097 ± 18.035	139.433 ± 33.707	148.975 ± 24.443	0.192	0.617
C20:5 *n*−3 (EPA)	15.563 ± 7.039	16.761 ± 8.005	15.812 ± 8.577	16.580 ± 7.211	0.025	0.644
C22:1	0.609 ± 0.201	0.583 ± 0.146	0.612 ± 0.286	0.660 ± 0.139	0.112	0.637
C22:5 *n*−3	6.968 ± 2.003	7.380 ± 1.419	6.786 ± 2.194	7.303 ± 1.606	0.011	0.641
C22:5 *n*−6	2.353 ± 0.613	2.476 ± 0.436	2.307 ± 0.880	2.533 ± 0.784	0.079	0.618
C22:6 *n*−3 (DHA)	43.766 ± 18.081	47.528 ± 18.181	46.919 ± 23.502	48.283 ± 21.276	0.059	0.493

**Table 5:** Free fatty acids compared to total fatty acids in RBCs at rest (Time point 1; *n* = 6)

Fatty acid (*μ*g/g)	Free fatty acids in RBCs	Total fatty acids in RBCs	Percentage of free fatty acids (%)
C12:0	0.135 ± 0.043	1.033 ± 1.677	13.1
C14:0	0.505 ± 0.254	16.296 ± 12.774	1.99
C14:1	0.027 ± 0.014	0.965 ± 0.230	2.8
C16:0	3.020 ± 0.221	551.129 ± 158.224	0.5
C16:1	0.232 ± 0.154	16.427 ± 6.420	1.4
C18:0	7.420 ± 1.697	349.925 ± 102.151	2.1
C18:1 cis	1.615 ± 0.662	369.359 ± 109.595	0.4
C18:1 trans	0.141 ± 0.083	11.990 ± 3.208	1.2
C18:2	0.508 ± 0.291	378,376 ± 1,172,868	1.3
C18:3 *n*−3	0.150 ± 0.149	8.298 ± 2.8805	1.8
C18:3 *n*−6	0.018 ± 0.012	3.436 ± 1.1632	0.5
C20:1	0.055 ± 0.048	12.580 ± 4.273	0.4
C20:3 *n*−6	0.025 ± 0.018	73.136 ± 32.1532	3.4
C20:4 *n*−6	0.278 ± 0.098	442.260 ± 116.511	6.2
C20:5 *n*−3 (EPA)	0.037 ± 0.005	21.916 ± 5.707	1.3
C22:1 *n*−9	0.010 ± 0.011	0.810 ± 0.329	1.2
C22:5 *n*−3	0.673 ± 0.542	29.426 ± 5.518	2.3
C22:5 *n*−6	0.298 ± 0.330	7.454 ± 2.019	3.9
C22:6 *n*−3 (DHA)	0.085 ± 0.110	145.677 ± 57.864	5.8

**Table 6:** Free fatty acids in RBCs in response to exercise (*n* = 6)

Fatty acid (*μ*g/g)	Time point 1 (rest)	Time point 3 (exhaustion)	t‐test, *P* value
C12:0	0.135 ± 0.043	0.127 ± 0.049	0.76
C14:0	0.505 ± 0.254	0.405 ± 0.108	0.39
C14:1	0.027 ± 0.014	0.022 ± 0.012	0.51
C16:0	3.020 ± 0.221	2.945 ± 0.275	0.614
C16:1	0.232 ± 0.154	0.135 ± 0.014	0.16
C18:0	7.420 ± 1.697	7.658 ± 1.929	0.82
C18:1 cis	1.615 ± 0.662	1.297 ± 0.328	0.32
C18:1 trans	0.141 ± 0.083	0.080 ± 0.028	0.11
C18:2	0.508 ± 0.291	0.363 ± 0.080	0.27
C18:3 *n*−3	0.150 ± 0.149	0.087 ± 0.041	0.34
C18:3 *n*−6	0.018 ± 0.012	0.013 ± 0.005	0.36
C20:1	0.055 ± 0.048	0.035 ± 0.014	0.35
C20:2 *n*−6	0.023 ± 0.023	0.017 ± 0.008	0.52
C20:3 *n*−6	0.025 ± 0.018	0.017 ± 0.005	0.29
C20:4 *n*−6	0.278 ± 0.098	0.225 ± 0.033	0.23
C20:5 *n*−3 (EPA)	0.037 ± 0.005	0.030 ± 0.006	0.07
C22:1 *n*−9	0.010 ± 0.011	0.007 ± 0.005	0.51
C22:5 *n*−3	0.673 ± 0.542	0.432 ± 0.109	0.29
C22:5 *n*−6	0.298 ± 0.330	0.160 ± 0.039	0.33
C22:6 *n*−3 (DHA)	0.085 ± 0.110	0.043 ± 0.012	0.38
C24:1 *n*−9	0.127 ± 0.134	0.098 ± 0.053	0.64

## Discussion

To our knowledge, this study is the first to assess the impact of acute exercise on individual RBC fatty acids. RBC fatty acid profiling was performed on venous blood taken form healthy subjects undergoing maximal treadmill exercise testing using the standard Bruce protocol, which is used in clinical routine as cardiac stress test to induce strong and robust increases in heart rate and blood pressure (Bruce et al. 1973; Trappe and Lollgen 2000). Although we did not confirm our hypothesis that RBC fatty acids, including the omega‐3 quotient, vary during exercise and/or post‐exercise (with exception of RBC lauric acid), we observed significant increases in heart rate, blood pressure and lactate in the volunteers at maximal exercise, which occurred at 13.5 METs. Furthermore, the omega‐3 quotient did also not vary during maximal exercise in healthy volunteers. Finally, short‐term exhaustive exercise did not induce increased mobilization of individual free fatty acids into plasma or greater rate of oxidation of the free fatty acids removed from the circulating blood during exercise.

### Omega‐3 fatty acids

The clinical impact of the omega‐3 quotient is well‐established. Omega‐3 index is associated with increased insulin sensitivity and more favorable metabolic profile in middle‐aged overweight men (Albert et al. 2014) and obese children (Burrows et al. 2011). A higher omega‐3 index is associated with older age, increasing body mass index, and a history of smoking and fish oil intake in patients with peripheral artery disease (Nosova et al. 2014). RBC *n*−6 fatty acids have been found to be positively associated, and RBC *n*−3 fatty acids are inversely associated with body weight gain (Wang et al. 2016). Interestingly, we did not detect changes in alpha‐linolenic acid, which is inversely related to development of adiposity, at least in school‐age children (Perng et al. 2015). Dietary omega‐3 fatty acids modulate the eicosanoid profile in man primarily *via* the CYP‐epoxygenase pathway, which could function as mediators of the vasodilatory and cardioprotective effects of omega‐3 fatty acids (Fischer et al. 2014). Recent results demonstrate that CYP enzymes efficiently convert EPA and DHA to novel epoxy and hydroxy metabolites that could mediate some of the beneficial cardiovascular effects of dietary omega‐3 fatty acids (Arnold et al. 2010). In recent randomized, double‐blind, placebo‐controlled trials, dietary *n*−3 fatty acid supplementation (3–6 g daily) mitigated the course of coronary atherosclerosis in humans (von Schacky et al. 1999), but had (1 g daily) no cardiovascular benefit in initially healthy adults and in patients with diabetes mellitus (Group et al. 2018; Manson et al. 2018). Nevertheless, recent data show that dietary EPA (4 g daily, REDUCE‐IT trial) is effective for prevention of major coronary events in hypercholesterolaemic patients (Yokoyama et al. 2007) and cardiovascular events in patients with established cardiovascular disease or with diabetes and other risk factors (Bhatt et al. 2018). Our results support the concept that the omega‐3‐ quotient is strongly affected by diet, e.g. DHA/EPA‐rish fish oil, but not acute maximal exercise.

### Omega‐9 fatty acids

High levels of omega‐9 monounsaturated fatty acids, oleic acid (OA, C18:1 cis), gondoic acid (GA, C20:1), and nervonic acid (NA, C24:1) in RBCs showed increased all‐cause and cardiovascular mortality in the Ludwigshafen Risk and Cardiovascular Health Study (Delgado et al. 2017). High levels of palmitic acid (C16:0) palmitoleic acid (C16:1), oleic acid (C18:1), and alpha‐linolenic acid (C18:3 *n*−3) were also associated with higher risk of sudden cardiac death (Lemaitre et al. 2009, 2010). Our study revealed a tendency towards elevated levels of the fatty acids C16.0, C16:1, C18:1, and C18:3 *n*−3 in RBC at exhaustion, but the effects were not statistically significant. Future studies are warranted to explore biologic and prognostic properties of omega‐9 fatty acids in long‐term effects on cardiovascular disease.

### Lauric acid

Lauric acid (C12:0) is the primary fatty acid found in coconut oil. High‐coconut Nourish and high‐virgin coconut oil diets improve cardiovascular and liver complications in obesity in rats (Panchal et al. 2017). RBCs have at least two classes of fatty acid binding sites for laurate and possibly other medium‐chain fatty acids (Spector et al. 1972). The stronger class has approximately 1.2 × 10^5^ sites/cell and an association constant for laurate of 1.8 × 10^6^ mol/L. These strong sites probably bind much of the “nonexchangeable” or “irreversibly bound” free fatty acid pool of the cell. The large, weaker class of binding sites contains approximately 8.0 × 10^6^ sites/cell and has an association constant for laurate of 1.3 × 10^4^ mol/L. The fatty acid present at these sites probably represents the “exchangeable” or “reversibly‐bound” free fatty acid pool of the cell (Spector et al. 1972). The observed post exercise decrease in RBC lauric acid (C12:0) could contribute to gain access to the lipid energy reserves in skeletal muscle, liver, and heart (Di Paola and Lorusso 2006; Exercise‐induced 2017), where medium‐chain fatty acids appear to be preferentially oxidized in mitochondria compared to long‐chain fatty acids. Since circulating C12:0 plasma levels and free C12:0 levels in RBCs were not affected by exercise, the decrease of total RBC lauric acid is possibly caused from its “nonexchangeable” or “irreversibly bound” free fatty acid pool in the red blood cells. The functional importance and downstream effects of lauric acid in metabolic and hemodynamic post exercise recovery remain to be determined.

### Exercise protocol considerations

The modified Bruce protocol was selected to ensure that all subjects were able to complete a similar highest intensity workload (13.50 ± 1.97 METs) concomitant with robust and significant short‐term increases in heart rate and blood pressure without fatiguing. For consistency, we used also an intermediate workload (10.00 ± 1.90 METs, *P *=* *0.0009, *t*‐test) reaching a heart rate of 150 or more beats per min to ensure that all runners could complete the test with marked increases in heart rate and blood pressure without fatiguing. Our clinical data show that the exercise protocol caused the expected hemodynamic and metabolic changes in the subjects under study.

## Limitations

We obtained venous blood from an arm vein, although the source free and RBC fatty acids, if modified from exercising muscle and its vasculature, suggests that the leg would provide a different measure. However, blood samples were taken from the arm vein because of the great difficulty associated with obtaining blood from a vein in dynamically contracting leg muscles. Therefore, stronger effects may have been present in the venous effluent of the exercising muscle (Giordano et al. 2011). We studied effects of maximal short‐term exercise, but not endurance exercise, which may also lead to different results. Interestingly, elite athletes have an increased incidence of sudden death and low omega‐3‐quotients (von Schacky et al. 2014). However, it is unknown whether this deficiency in omega‐3‐quotient results from reduced diet or extreme endurance exercise training.

## Conclusions

Our results suggest that dynamic exercise does not change the levels of RBC *n*−3 fatty acid status in the systemic circulation. Our results are consistent with the idea that the essential fatty acids (*n*−3 and *n*−6) are strongly affected by diet (Fischer et al. 2014), but not short‐term maximal exercise, whereas changes in RBC lauric acid, which can be synthesized *de novo* and is bound in red blood cells, reflects metabolic processes. More research is needed to determine the contribution of RBC fatty acids to cardiac performance and regulation of coronary and/or skeletal‐muscle blood flow in health and cardiovascular disease.

## Author Contributions

BG, MG, and FCL planned and designed the experimental studies. BG conducted and guided the treadmill experiments. ID and MR performed the HPLC‐MS spectrometry experiments. All authors contributed to the implementation and analyses of the experiments. BG drafted the article, and all authors, contributed to its completion.

## Conflict of Interest

None declared.
